# Reintroducing the Concept of Complementarity into Psychology

**DOI:** 10.3389/fpsyg.2015.01822

**Published:** 2015-11-27

**Authors:** Zheng Wang, Jerome Busemeyer

**Affiliations:** ^1^School of Communication, The Ohio State University, Columbus, OH, USA; ^2^Department of Psychological and Brain Sciences, Indiana University, Bloomington, IN, USA

**Keywords:** quantum cognition, quantum probability, complementarity, commutativity, compatibility, Niels Bohr, William James, order effects

## Abstract

Central to quantum theory is the concept of complementarity. In this essay, we argue that complementarity is also central to the emerging field of quantum cognition. We review the concept, its historical roots in psychology, and its development in quantum physics and offer examples of how it can be used to understand human cognition. The concept of complementarity provides a valuable and fresh perspective for organizing human cognitive phenomena and for understanding the nature of measurements in psychology. In turn, psychology can provide valuable new evidence and theoretical ideas to enrich this important scientific concept.

## Introduction

Central to quantum theory is the concept of complementarity. This essay argues that complementarity is also central to the emerging field of quantum cognition (e.g., Aerts and Aerts, 1994; Khrennikov, 1999; Pothos and Busemeyer, 2013; Wang et al., 2013; Bruza et al., 2015; Busemeyer and Wang, 2015), which applies abstract, mathematical principles of quantum theory to shed light on cognitive structures and processes. The concept of complementarity provides a valuable and fresh perspective for organizing human cognitive phenomena and for understanding the nature of measurements in psychology. In turn, psychology can provide useful new evidence and theoretical ideas to enrich this important scientific concept.

## Complementarity, Commutativity, and Compatibility

The general concept of complementarity was developed by Niels Bohr in a series of debates with Einstein, but the main idea can be summarized as follows (Plotnitsky, 2014, p. 5): Different measurement conditions for observing different phenomena are complementary when

(a) they are mutually exclusive, and only one can be applied at any time; and
(b) they are all necessary for a comprehensive account of these phenomena.

An important consequence of complementarity is that the sequence or order of the measurements matters (von Neumann, 1932, 1962; Atmanspacher and Römer, 2012; Wang et al., 2014). The above definition of complementarity is deliberately general so that it can permit many specific implementations. Below, we provide a way to implement this idea in psychology.

The essential idea of complementarity can be illustrated using the following example involving the measurements of attitudes toward politicians. In a 1997 poll in the United States, half of the 1,002 nationally sampled respondents were asked, “Do you generally think Clinton is honest?” Then they were asked the same question about Gore. The other half answered the same questions in the opposite order. The results exhibited a striking order effect: The proportion saying “yes” to both questions was significantly higher when Gore was judged first (Moore, 2002).

In this example, the phenomena of interest concern a survey respondent’s beliefs about the honesty of different politicians. Complementarity arises when a person cannot have a well-defined position on each politician simultaneously. We can obtain a measurement of honesty concerning Clinton or concerning Gore, but we cannot measure both simultaneously, and the order in which we measure them affects the answers. Once we obtain a measurement on say, Clinton, that decision can create a definite position for Clinton, but then the opinion regarding the Gore must be uncertain. However, both measurements are needed to obtain a complete understanding of a respondent’s attitude to the two politicians being considered. Therefore, these measurements satisfy the general requirements for complementarity.

This example captures another idea relevant to complementarity. The phenomena that we observe are products of an interaction between some object of investigation and our measurement instruments. The measurement does not simply record a phenomenon, but it creates one. This idea is consistent with the constructionist view of beliefs, attitudes, and intentions proposed by many psychologists (e.g., Feldman and Lynch, 1988; Schwarz, 2007). From this view, because of limited mental capacity and cognitive economy, beliefs, attitudes, and intentions do not exist in memory as properties ready to be recorded; instead, they are constructed when needed. When one is asked a subsequent question, information carried over from the preceding question provides a context for the construction of the second and influences the subsequent response.

Next, we will explain complementarity more specifically by providing a simple “toy” quantum model for this example. To do this, we need to first compare some concepts from classical and quantum probability theories (see Busemeyer and Bruza, 2012, for more detail).

**FIGURE 1:**
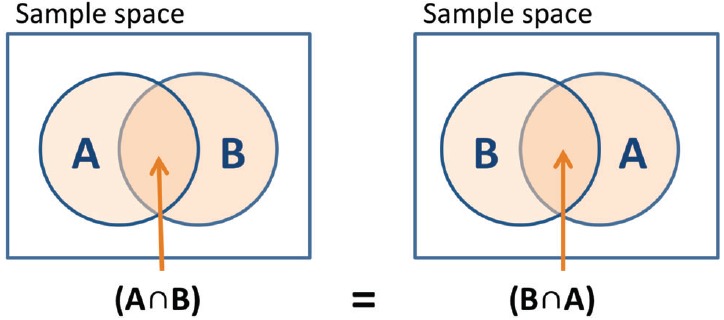
**Sets representation of events.** Classical probability theory has difficulty explaining order effects because events are represented as sets and are commutative, so the joint probability of events A and B is the same for the order of “A and B” and the order of “B and A.”

Classical probability theory is concerned with the assignment of probabilities to events. Suppose, for example, we ask a survey participant to evaluate various politicians with regard to their honesty. For example, an event, A, might be that politician X is evaluated as honest. According to classical probability theory, events are represented as subsets of a universal set^1^. For example, the event that a politician is honest is a subset of the universe of all the features that a politician might have. Another event, B, might be that politician Y is evaluated as dishonest. The conjunction of two events is defined by set intersection—in this case, A and B. As shown in Figure 1, the combined event “A and B” is the same as the combined event “B and A,” and therefore the order of the two events does not matter. Formally, we say that the intersection event is *commutative*, and the probability assigned to “A and B” must equal the probability assigned to “B and A.”

Quantum probability theory is also concerned with the assignment of probabilities to events. However, according to quantum theory, events are represented as subspaces of a universal vector space^2^. If events are defined as subspaces, then the conjunction of two events may or may not exist. The conjunction does not exist if the events are non-commutative so that the order of evaluating them matters. Events that are commutative are also called *compatible*, and events that are non-commutative are called *incompatible* (Atmanspacher and Römer, 2012). Classical probability theory essentially assumes that all events are compatible, but quantum probability theory allows some events to be incompatible.

**FIGURE 2:**
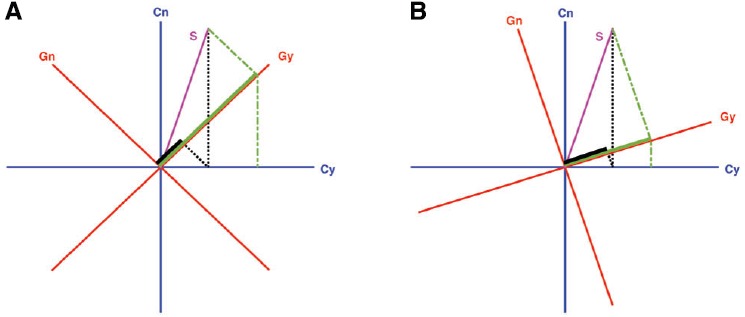
**A “toy” example of quantum probability model of sequential judgments. (A)** Illustrates how quantum model provides a natural account for question order effects of the Clinton-Gore example in terms of incompatibility (Wang and Busemeyer, 2013; Wang et al., 2014). First, consider the probability of a person’s answering “yes” to both questions when Clinton is judged first. This is obtained by first projecting (following the black dotted lines) the magenta-colored state *S* to the blue Cy (“yes” to Clinton) axis and then projecting the result up to the red Gy (“yes” to Gore) axis, which produces a small probability (as illustrated by the short length of the black projection on the Gy axis). Next, consider the probability of a person’s answering “yes” to both questions when Gore is judged first. This is obtained by first projecting (following the green dash-dotted lines) the state *S* down to the red Gy (“yes” to Gore) axis and then projecting the result to the blue Cy (“yes” to Clinton) axis, which produces a much higher probability. In addition, note that the probability of saying “yes” to Gore in this Gore-Clinton order is much higher than it is in the Clinton-Gore order (as illustrated by the long length of the green projection on the Gy axis), producing the order effect for the Gore question when the question is asked before, as opposed to after, the Clinton question. **(B)** Illustrates similar order effects, but the order effects are much smaller because the basis vectors (i.e., the red and blue axes), which form the subspace for evaluating the Gore and Clinton questions, are more aligned with each other. This means that the rotation required to change from one evaluation basis (e.g., the blue Clinton axes) to the other (e.g., the red Gore axes) is smaller. In a sense, in psychology we could understand this as “reduced incompatibility” of the two questions, or the two sets of projectors as “more nearly commutative,” although in quantum physics, the original concept of complementarity does not have the notion of degrees of complementarity (events can be differentiated only by being complementary or not).

Figure 2 illustrates how the projective geometry used by quantum probability theory naturally accounts for order effects. The “yes” answer to the “Do you generally think Clinton is honest?” question is represented by the horizontal ray (which forms one axis from the blue basis), and the “yes” answer to the “Do you generally think Gore is honest?” question is represented by an oblique ray (which forms one axis from the red basis). These two answers are *incompatible* because the subspaces (rays in this “toy” example) for these answers are not defined by a common basis. A person needs to evaluate the Clinton question using one pair of axes (the blue axes), and then must shift her or his viewpoint to another pair of axes (the red axes) to evaluate the Gore question. The final result depends on the order of the applications, because answering one question provides a new contextualized state that is used to generate responses to the second question. As a consequence of incompatibility, if a person is certain about an answer to one question, then the person must be uncertain about the answer to the other question (evidencing the uncertainty principle of quantum theory). In other words, when the questions are incompatible, one cannot be certain about the answers to both questions simultaneously (evidencing the superposition principle of quantum theory).

A key point here is that different bases (red vs. blue axes in Figure 2) are required to perform the Clinton and Gore measurements. According to quantum theory, two measurement conditions are complementary whenever we have to change the basis used to represent the outcomes of each measurement.

*What makes two measures compatible in psychology?* Two questions are compatible if the subspaces representing each question are defined by a common basis. In our example, to form a common basis for representing the Clinton and Gore questions, we must posit at least a four-dimensional space, with the four basis vectors (or axes) representing the four conjunctions: (1) “yes” to Clinton and “yes” to Gore, (2) “yes” to Clinton and “no” to Gore, (3) “no” to Clinton and “yes” to Gore, and (4) “no” to Clinton and “no” to Gore. The belief state would be a vector in this four-dimensional space, and each coordinate would indicate the belief about a conjunction (e.g., the belief in “yes” to Clinton and “no” to Gore). When a compatible representation is used, the order of questions does not matter, because the person eventually arrives at the same conjunction with the same probability when finished. Also, the person can be certain about the answers to both questions at the same time. This seems like a more ideal case of human cognition. This, however, all comes at a higher cost, because more cognitive resources are needed to increase and maintain the higher dimensionality of the compatible representation space (Wang and Busemeyer, 2013; Bruza et al., 2015).

## From Psychology to Physics: The History

It is an interesting twist of the history that the term “complementary” first appeared in the foundational work of psychology. In one of the most influential classic works in psychology, *The Principles of Psychology*, James (1890) wrote,

“…in certain persons, at least, the total possible consciousness may be split into parts which coexist but mutually ignore each other, and share the objects of knowledge between them. More remarkable still, they are complementary.” (p. 204)

Although there is still debate among philosophers and historians whether Bohr’s concept of complementarity was influenced by James, many agree on the clear similarity between the concept of complementarity that James created for psychology in 1890 and that Bohr introduced into physics four decades later, and believe Bohr was at least indirectly affected by James’s work (e.g., Stapp, 1993; Plotnitsky, 2012). The concept of complementarity emerged around 1926 and 1927 from the discussions between Bohr and Werner Heisenberg related to the discovery of the uncertainty principle. In a lecture in Como, Italy, in 1927, Bohr (1928) for the first time discussed complementarity in public, and the lecture was published the next year. By the time of the famous debate with Einstein regarding the Einstein-Podolsky-Rosen experiment, Bohr had developed a rather complete definition of complementarity (Plotnitsky, 2014):

“Evidence obtained under different experimental conditions cannot be comprehended within a single picture, but must be regarded as complementary in the sense that only the totality of the phenomena exhaust the possible information about the objects…” (Bohr, 1987a, p. 40)

It is interesting that as an adolescent, Bohr had shown interest in describing human conscious processes (Folse, 1985, p. 175). Even in his earlier papers on complementarity and quantum physics, he tried to state how the concept of complementarity could be applied to psychology. For example, he ended his Como lecture,

“I hope, however, that the idea of complementarity is suited to characterize the situation, which bears a deep-going analogy to the general difficulty in the formation of human ideas, inherent in the distinction between subject and object.” (Bohr, 1928, p. 590)

A year later, in a paper he wrote for a Planck *Festschrift* in 1929, he stated his view on applying complementarity to psychology with greater clarity:

“For describing our mental activity, we require, on one hand, an objectively given content to be placed in opposition to a perceiving subject, while, on the other hand, as is already implied in such an assertion, no sharp separation between object and subject can be maintained, since the perceiving subject also belongs to our mental content. From these circumstances follows not only the relative meaning of every concept, or rather of every word, the meaning depending upon our arbitrary choice of view point, but also that we must, in general, be prepared to accept the fact that a complete elucidation of one and the same object may require diverse points of view which defy a unique description. Indeed, strictly speaking, the conscious analysis of any concept stands in a relation of exclusion to its immediate application. The necessity of taking recourse to a complementary, or reciprocal, mode of description is perhaps most familiar to us from psychological problems.” (Bohr, 1987b, p. 96)

Complementarity is not limited to physics. Instead, it is a general concept that can be applied to any phenomena that are featured by “a participating observer.” As Bohr recognized, these kinds of phenomena are typical in psychology. In the end, psychology is the field that studies “the participating observer”—the observer’s perception, attention, emotion, motivation, memory, and decision-making, among other psychological processes.

The concept of complementarity applies naturally to psychological systems. Just like a physical system, a psychological system can be measured in different, mutually exclusive ways. Although all these measurements are essential for describing the system, they cannot be measured simultaneously, only sequentially. In this case, we say the different measurements are complementary (Stapp, 1993). Importantly, this means that the measurement is “an essential part of making a property definite” (Stapp, 1993, p. 234). In other words, measurements do not merely record the property of a system but construct it.

## Empirical Testability of Complementarity in Psychology

Two criticisms are often raised in response to quantum cognition because of misunderstandings of this new research program. One we believe is a false alarm due to a general resistance to—and often a legitimate concern about—the loose, vague, metaphorical, speculative extension of quantum physics to cultural and social studies (Beller, 1998). However, differently from what is being argued against, the research program of quantum cognition rigorously uses mathematical principles of quantum probability theory to build new models of human cognition, develop specific new predictions, and empirically test the new predictions and compare new models against existing traditional models. Just like other cognitive models based on classical probability theory, quantum cognition models take advantage of quantum formalism to provide new theoretical and modeling tools that make precise predictions regarding human cognition.

The other typical criticism questions whether quantum cognition can ever provide the kind of rigor and precision that is shown by quantum mechanics. Unfortunately, it is true that compared to quantum physics, which provides rigorous and precise predictions about physical phenomena, psychological theories involve many more random variables that are hardly controlled, resulting in lower precision in prediction. To be fair, this is a general challenge for any theories in the behavioral and social sciences. However, through rigorous model comparison, empirical studies have shown that quantum models provide an elegant new way to specify general and vague verbal theories in psychology, and better explain and predict many phenomena puzzling to classical models, leading to highly testable models (e.g., Bruza et al., 2015; Busemeyer and Wang, 2015; Busemeyer et al., 2015).

In fact, compared to many other psychological theories and models, quantum cognitive models may be more falsifiable. Because quantum cognitive models are based on a coherent set of axioms that are clearly stated, these models must stand up to strict tests of these axioms in addition to performance comparisons against competing classical models. Using our quantum question order model as an example again, the model provides clear theoretical predictions about when order effects will or will not occur as well as the pattern of order effects that do occur (Wang and Busemeyer, 2013). One of the most convincing examples illustrating the testability of quantum models has been an *a priori*, parameter-free, and precise test called the quantum question equality, or QQ equality (Wang and Busemeyer, 2013; Wang et al., 2014). This equality, derived from quantum theory, imposes a strong symmetry condition on the nature of order effects, and empirical results from more than 70 U.S. national surveys provided surprisingly strong support for this precise prediction (Wang et al., 2014). Rarely in social science research do we find *a priori* and parameter-free predictions being upheld with such high accuracy. Classical models cannot explain—in a principled and *a priori* manner—both the question order effects and the QQ equality observed in the empirical data (Wang et al., 2014).

## Extending the Concept of Complementarity in Psychology

Psychology provides an opportunity to extend and enrich the concept of complementarity beyond what is being formulated in physics. When applied to psychology as opposed to physics, compatibility may take on a more fluent and malleable role. Perhaps compatibility varies across individuals, develops across age, and changes with experience. For example, very young children do not seem to have the ability to take on the perspective of another person—this capability to change perspectives develops only after a critical developmental stage (e.g., Epley et al., 2004).

As another example, perhaps compatibility can be formed after an individual has had many experiences with combinations of events that permit the formation of conjunctive concepts. To be more specific, if a combination of questions is new or unusual, then an answer must be constructed on-line that relies on a simpler, incompatible, lower-cost representation. However, if a person has a great deal of experience with a combination, then the person may have sufficient knowledge to form a compatible representation as a result of cognitive adaptation to the environment. Therefore, order effects are expected to occur for uncommon or unfamiliar pairs of questions, whose answers must be (at least partially) constructed on the spot. Indeed, two field experiments during the 1988 and 1992 presidential elections supported this possibility (Simmons et al., 1993). The authors found that the question order effects on issue opinions decreased as the election became closer, which would be predicted by the quantum model because the measurements on issue opinions might more frequently occur over time during media exposure or daily conversations—even if the measurements were not directly noted.

In sum, at this early stage of research, the concept of compatibility is new in psychology, and we can only speculate about which measures will be compatible or incompatible. Then the speculations or assumptions can be empirically tested based on order effects or interference effects of the measures, among other predicted effects that follow incompatibility. However, this will be a crucial question for future research in quantum cognition, which should enrich the concept of complementarity through psychological experiments and theories.

## Concluding Comments

As we have described, the idea of complementarity was introduced into psychology by James (1890). Later, the idea was developed formally and became one of the centerpieces of Niels Bohr’s interpretation of quantum mechanics. Unfortunately, for many years the concept appeared to be useful only in physics, and it almost disappeared from the psychological literature (for exceptions, see Grossberg, 2000). In this article, we have attempted to reintroduce the concept of complementarity to its original home in psychology. We think the concept provides an invaluable service toward understanding the fundamental nature of human cognition.

### Conflict of Interest Statement

The authors declare that the research was conducted in the absence of any commercial or financial relationships that could be construed as a potential conflict of interest.

## Footnotes

1 The universal set is a set that contains a sigma algebra of subsets. The subsets are the events that can occur and the events are subsets of the universal set.

2 The universal vector space is a vector space spanned by a set of basis vectors. The vector space contains subspaces, which are closed subsets of this vector space, and events are subspaces of the vector space.
